# Genome-Wide Association Study for Pre-harvest Sprouting Resistance in a Large Germplasm Collection of Chinese Wheat Landraces

**DOI:** 10.3389/fpls.2017.00401

**Published:** 2017-04-06

**Authors:** Yong Zhou, Hao Tang, Meng-Ping Cheng, Kwame O. Dankwa, Zhong-Xu Chen, Zhan-Yi Li, Shang Gao, Ya-Xi Liu, Qian-Tao Jiang, Xiu-Jin Lan, Zhi-En Pu, Yu-Ming Wei, You-Liang Zheng, Lee T. Hickey, Ji-Rui Wang

**Affiliations:** ^1^Triticeae Research Institute, Sichuan Agricultural UniversityChengdu, China; ^2^Ministry of Education Key Laboratory for Crop Genetic Resources and Improvement in Southwest China, Sichuan Agricultural UniversityYa’an, China; ^3^Queensland Alliance for Agriculture and Food Innovation, The University of Queensland, BrisbaneQLD, Australia

**Keywords:** wheat, landrace, pre-harvest sprouting, GWAS, haplotypes, geographic distribution

## Abstract

Pre-harvest sprouting (PHS) is mainly caused by the breaking of seed dormancy in high rainfall regions, which leads to huge economic losses in wheat. In this study, we evaluated 717 Chinese wheat landraces for PHS resistance and carried out genome-wide association studies (GWAS) using to 9,740 DArT-seq and 178,803 SNP markers. Landraces were grown across six environments in China and germination testing of harvest-ripe grain was used to calculate the germination rate (GR) for each accession at each site. GR was highly correlated across all environments. A large number of landraces (194) displayed high levels of PHS resistance (i.e., mean GR < 0.20), which included nine white-grained accessions. Overall, white-grained accessions displayed a significantly higher mean GR (42.7–79.6%) compared to red-grained accessions (19.1–56.0%) across the six environments. Landraces from mesic growing zones in southern China showed higher levels of PHS resistance than those sourced from xeric areas in northern and north-western China. Three main quantitative trait loci (QTL) were detected by GWAS: one on 5D that appeared to be novel and two co-located with the grain color transcription factor *Tamyb10* on 3A and 3D. An additional 32 grain color related QTL (GCR-QTL) were detected when the set of red-grained landraces were analyzed separately. GCR-QTL occurred at high frequencies in the red-grained accessions and a strong correlation was observed between the number of GCR-QTL and GR (*R*^2^ = 0.62). These additional factors could be critical for maintaining high levels of PHS resistance and represent targets for introgression into white-grained wheat cultivars. Further, investigation of the origin of haplotypes associated with the three main QTL revealed that favorable haplotypes for PHS resistance were more common in accessions from higher rainfall zones in China. Thus, a combination of natural and artificial selection likely resulted in landraces incorporating PHS resistance in China.

## Introduction

Pre-harvest sprouting (PHS) is defined as the germination of grains within mature spikes on the mother plant before harvest (Nyachiro, 2012). In wheat (*Triticum aestivum* L.), PHS is mainly caused by the breaking or lack of seed dormancy under humid and wet conditions, which leads to huge economic losses due to decreased grain weight and end-use quality (Zhang and Liu, 1989; Kulwal et al., 2012). Thus, seed dormancy (SD) has been considered the major factor that determines PHS resistance (Bewley and Black, 1982; Mares and Mrva, 2001; Finch-Savage and Leubner-Metzger, 2006). The world’s major wheat production regions, including Canada, Australia, and China, experience regular losses due to PHS (Rajjou et al., 2012). In China, PHS is a major abiotic constraint that reduces yield and production quality of wheat grain and has affected about 24.91 million ha of wheat fields (Xiao et al., 2002). Therefore, breeding for PHS-resistant cultivars is of great importance in China. The Chinese Academy of Agricultural Sciences (CAAS) has defined 10 wheat-growing zones in China, according to wheat type, varietal reactions to temperature, wheat-growing season and other factors (He et al., 2001). PHS is common in zones III-YTS (Middle and Low Yangtze Valleys Autumn-Sown Spring Wheat Zone), IV-SAS (Southern Autumn-Sown Spring Wheat Zone), V-SWAS (Southwestern Autumn-Sown Spring Wheat Zone), and VI-NES (Northeastern Spring Wheat Zone) (Jin, 1996; He et al., 2000; Xiao et al., 2002; Yuan et al., 2003; Liu L. et al., 2013).

A total of 110 quantitative trait loci (QTL) or loci associated with resistance to PHS in wheat have been reported in 24 previous mapping studies (Supplementary Table S1). These studies have either evaluated PHS resistance directly by testing whole intact spikes in misting chambers or simulated rain events in the field (Somyong et al., 2014; Albrecht et al., 2015), or germination testing of harvest-ripe grain under controlled conditions (Somyong et al., 2014; Zhang et al., 2014; Lin et al., 2015). According to biparental genetic linkage analyses, all 21 chromosomes of wheat reportedly harbor QTL for PHS resistance (Mohan et al., 2009; Cabral et al., 2014; Cao et al., 2016; Fakthongphan et al., 2016), but the most consistently detected regions are located on the group three chromosomes (Kato et al., 2001; Osa et al., 2003; Kulwal et al., 2004; Mori et al., 2005; Liu and Bai, 2010) and Chr 4A (Mares et al., 2005; Chen et al., 2008; Singh et al., 2010; Cabral et al., 2014). The PHS resistance genes underpinning the 3A, 3B, and 3D regions are considered to be tightly linked or pleiotropic with red seed coat color determined by dominant R alleles (Himi et al., 2011). Thus, red-grained wheat cultivars typically display superior levels of PHS resistance. However, the major QTL on Chr 4AL is not associated with grain color (Mares et al., 2005; Tan et al., 2006; Chen et al., 2008; Imtiaz et al., 2008; Ogbonnaya et al., 2008; Singh et al., 2010; Liu et al., 2011; Cabral et al., 2014) and the underlying casual gene for grain dormancy (MKK3) was recently cloned by Torada et al. (2016).

Several genome-wide association studies (GWAS) have also reported candidate loci associated with PHS resistance (Supplementary Table S1). Jaiswal et al. (2012) used 250 simple sequence repeat (SSR) markers to scan 242 common wheat accessions and identified 30 markers associated with PHS, including eight previously reported markers. Kulwal et al. (2012) scanned 198 white winter wheat accessions using 1,166 Diversity Array Technology (DArT) and SSR markers, and identified eight QTL on seven chromosomes, including a novel QTL on Chr 7BS. Rehman Arif et al. (2012) reported 70 DArT markers positioned on 11 chromosomes were associated with PHS and SD in a collection of 96 winter wheat cultivars. Albrecht et al. (2015) carried out GWAS for a panel of 124 European winter wheat accessions using DArT and SSR markers, and detected five QTLs on Chr 1B, 1D (two QTL), 3D, and 5D.

Chinese wheat landraces display higher PHS resistance than improved cultivars (Wang et al., 2011; Liu et al., 2014), thus present valuable genetic resources for identifying candidate loci associated with PHS resistance that could be used in modern breeding programs. In the present study, a collection of 717 wheat landraces from major wheat-growing zones in China were phenotyped for PHS resistance over 4 years (2012–2015) at three locations. In order to identify markers that are closely positioned to new or known candidate genes and QTL, the accessions were genotyped using high density DArT-seq and single nucleotide polymorphism (SNP) arrays. We investigate the frequency of favorable alleles for PHS resistance in landraces originating from different wheat-growing regions in China.

## Materials and Methods

### Chinese Wheat Landraces

**FIGURE 1:**
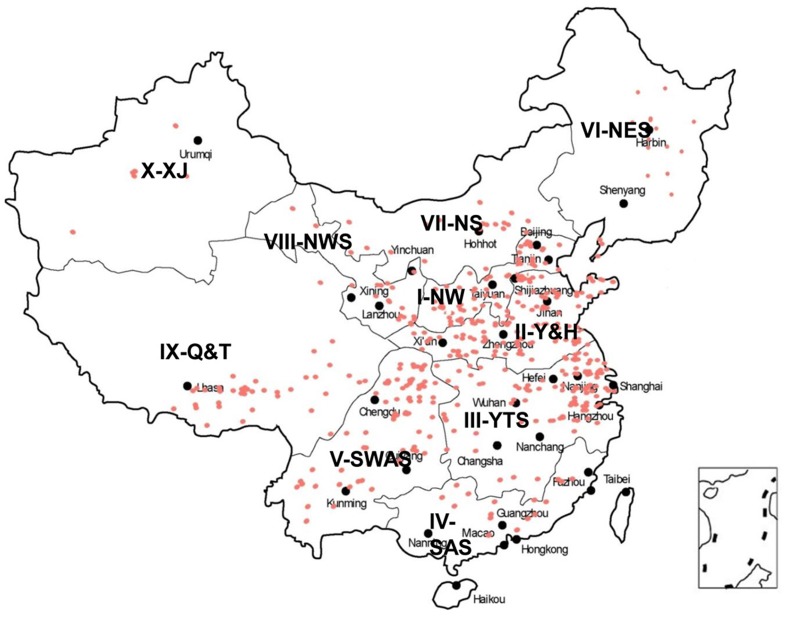
**Distribution of Chinese wheat landraces in 10 major agro-ecological production zones of China**.

Seven hundred and seventeen wheat landraces from 10 major wheat-growing zones in China were obtained from the CAAS (Supplementary Table S2 and **Figure 1**). The landraces were evaluated from 2012 to 2015 at experimental farms at Wenjiang (30°42′41.1′′N 103°52′06.7′′E), Chongzhou (30°32′39.9′′N 103°39′08.6′′E), and Ya’an (29°58′39.9′′N 102°59′21.9′′E) in Sichuan.

### Phenotyping for PHS Resistance

We evaluated PHS resistance by performing germination tests of harvest-ripe grain under controlled conditions (Somyong et al., 2014; Zhang et al., 2014; Lin et al., 2015). At each site, wheat spikes were harvested at physiological maturity (i.e., after loss of green pigmentation in the spikes and peduncles). The spikes were air-dried at room temperature for 7 days, avoiding direct sunlight and high temperature. Spikes were then stored at -20°C to preserve grain dormancy (Mares, 1983). Once samples of all accessions had been collected, threshing was performed by hand to aviod damaging the seed coat or embryos. Germination testing was conducted at 20 ± 1°C for 7 days and used three replicate petri dishes lined with filter paper for each accession, where each petri dish contained 50 grains. Germination was defined as the rupture of the grain coat by the emerging radicle. Germination was recorded over a period of 7 days and used to calculate cumulative percentage germination or germination rate (GR) to estimate the degree of SD (Osa et al., 2003; Mori et al., 2005; Torada et al., 2005). A GR apoproaching 100% indicates low levels of grain dormancy or PHS resistance (i.e., all grains germinated), whereas a GR approaching 0% indicates high levels of grain dormancy or PHS resistance. One-Way Analysis of variance (ANOVA) of GR for accessions derived from the 10 Chinese wheat-growing zones, which was performed by Duncan’s multiple test in each phenotyping tested environment. Two-tailed Pearson product-moment correlation coefficient tests were also carried out for GR obtained across the six environments (Fieller et al., 1957).

### Genotyping

The collection of 717 wheat landraces was genotyped using the DArT-seq (Diversity Arrays Technology, Canberra, ACT, Australia) genotyping-by-sequencing (GBS) platform. A subset of 272 landraces, that were representative of the genetic diversity, was also genotyped using the Axiom^®^ Wheat660 SNP array (Affymetrix^1^, Santa Clara, CA, USA). A total of 89,284 probes from the DArT-seq (DArT and DArT_GBS) and 630,517 probes from the Wheat660 SNP arrays were used for genotyping. Markers with maximum missing values of 10% were discarded and only those with minor allele frequency (MAF) ≥ 0.05 were used for further analyses.

### Genome-Wide Association Study (GWAS) for PHS Resistance

Compressed mixed linear model (Wang et al., 2005; Zhang Z. et al., 2010) accounting for the population structure (Pritchard, et al., 2000) and familial relationship (Li et al., 2013) was used to examine the association between markers and PHS phenotype using Tassel 4.0 (Bradbury et al., 2007). Population structure was assessed using the Bayesian clustering algorithm implemented by Structure 2.3.4 (Pritchard et al., 2000; Falush et al., 2003; Hubisz et al., 2009). An admixture model with 10 replicates for each number of genetic clusters (K, ranging from 1 to 10) and 10,000 iterations of burn-in followed by 10,000 MCMC iterations was used. The outputs of the genetic cluster analysis were extracted in STRUCTURE HARVESTER (Earl and vonHoldt, 2012) and the optimal alignment of the 10 iterations was determined using CLUMPP (Jakobsson and Rosenberg, 2007).

Four separated GWAS analyses were performed with four sets of STRUCTURE data in this study: (1) association analysis for 717 Chinese landrace wheat accessions using DArT-seq markers, (2) association analysis for 272 accessions using Wheat660 SNP markers, (3) association analysis for 77 white-grained accessions by Wheat660 SNP markers, and (4) association analysis for 186 red-grained accessions by Wheat660 SNP markers. GWAS sets 3 and 4 were conducted to explore the possibility of detecting QTL specific to the white- and red-grained germaplsm pools, termed grain colour releated QTL (GCR-QTL). The threshold for significant marker-trait associations was set at -log_10_ (0.01/*n*, where *n* = number of markers) for GWAS sets 1 and 2, and –log_10_ (0.001/n, where *n* = number of markers) for GWAS sets 3 and 4, which roughly equates to a Bonferroni correction (Weisstein, 2004; Su et al., 2016). Manhattan plots were generated using the qqman R package (Turner, 2014) in R i386 3.0.3 (R Core Team, 2014). Markers detected in at least two environments were used for QTL determine, and markers positioned with a 10 Mb region were considered the same QTL region. Markers associated with PHS resistance in this study were compared with QTL, loci and genes previously reported in the literature using a genetic map including 90K SNP, expressed sequence tag, SSR and DArT markers was reported by Cabral et al. (2014). The chromosomal locations of the 90K SNPs (Supplementary Table S7), DArT-seq markers and Wheat660 SNP markers were determined using the wheat ‘Chinese Spring’ survey sequence version 2.28^2^.

### Estimation of Haplotype Effects

Haplotype analyses were carried out for major QTL detcted in GWAS sets 1 and 2. Popart 1.7^3^ (Leigh and Bryant, 2015) was used to carry out haplotype analyses, and accessions with missing values were not included. The minimum spanning networks method was used to show the relationship between haplotypes and Median-joining networks for inferring intraspecific phylogenies (Bandelt et al., 1999). In each of the test environments, analysis of vairance (ANOVA) was conducted by taking genotypes as fixed effects and environments as random effect using SPSS version 16.0 (SPSS Inc., Chicago, IL, USA). ‘Favorable’ haplotpyes (allele and allelic combinations) were those that significantly lowered GR (increasing PHS resistance) compared to alternative haplotypes based on ANOVA. Thus, alterntive haplotypes were considered ‘unfavorable’ haplotpyes for PHS resistance. Accessions genotyped with the Wheat660 SNP array were used to determine the number of favorable haplotypes in each accession for haplotype pyramiding analysis. The frequency of favorable haplotpyes in the landrace collection was determined as the proportion of accessions that carried the favorable haplotpye.

## Results

### Variation for PHS Resistance in Chinese Wheat Landraces

**FIGURE 2:**
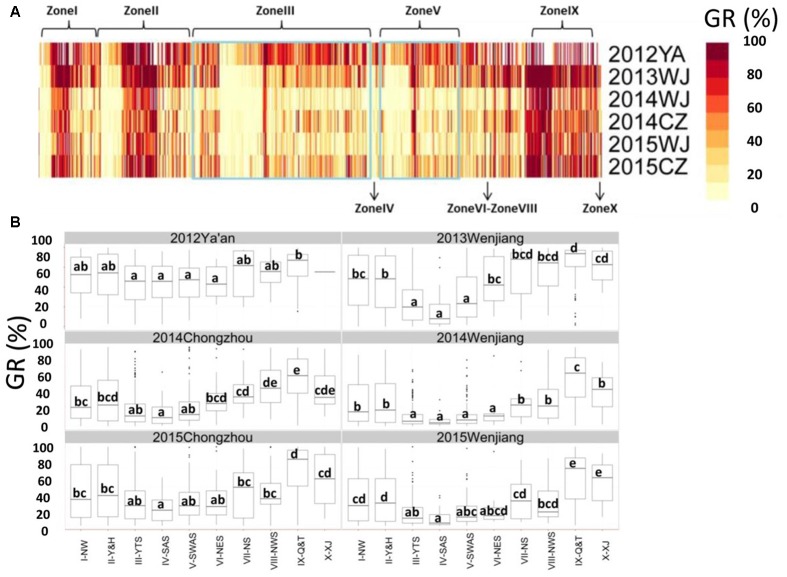
**Phenotypic analysis for germination rate (GR) obtained by Chinese wheat landraces. (A)** GR of Chinese wheat landraces grown from 2012 to 2015 at Wenjiang, Chongzhou, and Ya’an; **(B)** Box plot for GR obtained by accessions grouped according to origin (i.e., the 10 major agro-ecological production zones of China). The middle horizontal line within each box represents the mean GR and vertical lines mark the range from 5 to 95% of the total GR within each group.

Pre-harvest sprouting resistance was evaluated for a collection of 717 landraces grown at three locations (Chongzhou, Wenjiang, and Ya’an) from 2012 to 2015 (**Figure 1** and Supplementary Table S2). Most accessions displayed relatively stable phenotypes across the six environments (**Figure 2A**). The lowest mean GR (24.8%) was recorded in 2014 at Wenjiang, whereas the highest mean GR (60.8%) was recorded in 2012 at Ya’an (Supplementary Table S3). The GR was highly correlated across environments (*r* = 0.54–0.80) based on the two-tailed Pearson product-moment correlation coefficient test (Fieller et al., 1957) (**Table 1**). A total of 194 landraces exhibited a mean GR < 20.0%, of which 23 landraces displayed consistently dormant phenotypes across all six environments (Supplementary Table S2). Overall, white-grained accessions displayed significantly higher GR (mean range 47.2–79.6%) compared to red-grained accessions (mean range 19.1–56.0%) across all test environments (*p* = 8.52E-28–9.88E-13). Most landraces from wheat-growing zones III-YTS, IV-SAS, and V-SWAS, which are high rainfall regions in southern China, had red colored grain and displayed a significantly lower mean GR than landraces from other wheat-growing zones (**Figure 2B** and Supplementary Table S4). No accessions from zone IV-SAS were white-grained and only 8.5 and 7.9% of landraces from zones III-YTS and V-SWAS, respectively, produced white grains. Only a small number of landraces with white grains showed a mean GR < 20.0% across all six environments (i.e., Baitiaoyu, Baikangyangmai, Xiaoganmai, Baixu, Xiaoqingmang, Tuotuomai, Hechuanmai, Changxuxuqiaomai, and Dabaili). In contrast, 43.1 and 40.3% of landraces from zones I-NW and II-Y&H were red-grained and exhibited a significantly higher mean GR, respectively. Although higher percentages of accessions with red grains were from zones VIII-NWS and IX-Q&T, landraces from these two regions displayed lower levels of PHS resistance.

**Table 1:** Pearson product-moment correlation coefficient (PPCC) of germination rate among all measured environments.

Correlations	2012 Ya’an	2013 Wenjiang	2014 Wenjiang	2014 Chongzhou	2015 Wenjiang	2015 Chongzhou
2012 Ya’an						
2013 Wenjiang	0.56^∗∗^					
2014 Wenjiang	0.54^∗∗^	0.70^∗∗^				
2014 Chongzhou	0.55^∗∗^	0.68^∗∗^	0.69^∗∗^			
2015 Wenjiang	0.56^∗∗^	0.69^∗∗^	0.81^∗∗^	0.68^∗∗^		
2015 Chongzhou	0.62^∗∗^	0.68^∗∗^	0.74^∗∗^	0.68^∗∗^	0.77^∗∗^	

^∗∗^*Correlation is significant at the 0.01 level (2-tailed)*.

In order to investigate variation for PHS resistance associated with the origin of Chinese landraces, the mean GR obtained by accessions from each of the 10 zones were compared. Accessions from high rainfall zones III-YTS, IV-SAS, and V-SWAS showed a significantly lower mean GR than accessions derived from the other seven zones in at least three environments (**Figure 2B**). Further, accessions from zone IX-Q&T showed a significantly higher mean GR than accessions from other zones in at least three environments (**Figure 2B**). Accessions from zone IV-SAS displayed the lowest mean GR across all six environments (6.0–20.9%), however, this was similar to accessions from zones III-YTS (12.0–30.0%) and V-SWAS (13.1–36.9%). Accessions from zone VI-NES showed a significantly higher GR (15.5–58.8%) compared to accessions from zone IV-SAS in two of the six environments (i.e., Wenjiang in 2013 and Chongzhou in 2014). But accessions from zone VI-NES exhibited similar levels of PHS resistance with those from zones III-YTS and V-SWAS in all environments except Wenjiang in 2013. Accessions from zone I-NW (30.7–8.1%), II-Y&H (30.0–56.0%), VII-NS (29.4–66.0%), VIII-NWS (27.9–70.4%), IX-Q&T (58.9–80.8%), and X-XJ (35.7–70.7%) showed a significantly higher mean GR than accessions from zone IV-SAS in all test environments. Overall, accessions from zone IX-Q&T showed the highest mean GR compared to accessions from all other nine zones, but the mean GR was only deemed significantly higher in two of the six test environments. Overall, analysis of germination data using harvest-ripe grain collected from six environments revealed that wheat landraces originating from high rainfall zones in China (III-YTS, IV-SAS, and V-SWAS) displayed superior levels of PHS resistance.

### GWAS for PHS in Chinese Wheat Landraces

A total of 9,740 polymorphic markers with MAF ≥ 0.05 were selected from 89,284 DArT-seq markers for Bayes structure analysis and GWAS using 717 landrace accessions. Out of the 717 accessions, 272 that were representative of the genetic diversity were selected and genotyped using the Axiom^®^ Wheat660 SNP array (Supplementary Table S2). From the Wheat660 SNP array, 178,803 polymorphic SNP markers with MAF ≥ 0.05 were selected out of 630,517 total SNP markers. This subset of SNP markers were used for Bayes structure analysis and GWAS. Based on the genetic clusters analysis in Structure Harvester, *K* = 5 provided the highest peaks in both DArT-seq and Wheat660 data sets.

**FIGURE 3:**
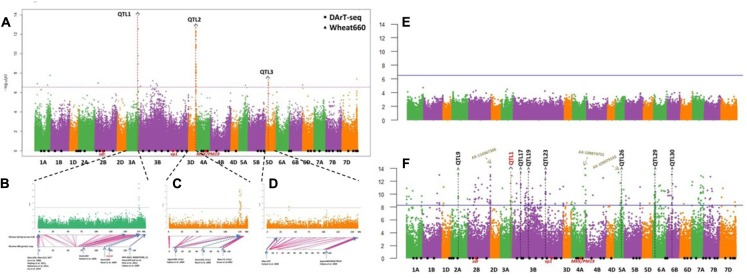
**Manhattan plots of genome-wide association studies (GWAS) results for pre-harvest sprouting (PHS) resistance in Chinese wheat landraces**. **(A)** Three main quantitative trait loci (QTL) identified by GWAS set 1 DArT-seq and 2 Wheat660; **(B)** QTL1 on Chr 3A and co-locating previously reported QTL; **(C)** QTL2 on Chr 3D and co-locating previously reported QTL; **(D)** QTL3 on Chr 5D and co-locating previously reported QTL; **(E)** GWAS results of GWAS set 3 77 white-grained accessions; **(F)** GWAS results of set 4 186 red-grained accessions.

Genome-wide association studies detected three highly significant DArT-seq and seven highly significant SNP markers. These markers satisfied the threshold for significance applied in this study; a –log_10_P value > 6.55 [-log_10_ (0.01/178803)] (Su et al., 2016) and were detected in at least two test environments. The chromosome position of these 10 highly associated markers revealed three main QTL regions that potentially contain PHS resistance genes (**Figure 3A**, **Table 2**, and Supplementary Table S5). QTL1 and QTL3 were represented by only one marker each, while QTL2 comprised eight markers. SNP marker AX-111578083 located at 173.81 Mb was linked with QTL1 on Chr 3A. This marker was detected in all environments with the exception of 2012Ya’an and explained 11.5–25.1% of the phenotypic variation. SNP marker AX-109028892 located at 39.36 Mb was linked with QTL 3 on Chr 5D. This marker was identified in three environments; 2013Wenjiang, 2015Wenajiang, and 2015Chongzhou and explained 11.5–12.0% of the phenotypic variation. The eight markers associated with QTL2 on Chr 3D consisted of three DArT-seq markers and five Wheat660 SNP markers. The three DArT-seq markers; A11134, A36351, and A36269 were located at 110.99 Mb, 111.54 Mb, and 113.89 Mb, respectively. They were detected in 4, 5, and 6 environments and explained 4.4–8.8% of the phenotypic variation, respectively. The five SNP markers; AX-111204246, AX-108879360, AX-111624595, AX-110772653, and AX-95124645 were located at 112.35 Mb, 112.36 Mb, 112.63 Mb, 112.80 Mb, and 113.74 Mb on Chr 3D and explained 11.7–19.3% of the phenotypic variation, respectively (**Table 2** and Supplementary Table S5). SNP markers AX-111204246 and AX-95124645 were associated in two environments, while AX-111624595 and AX-110772653 were associated in four environments, and AX-108879360 was associated in three environments.

**Table 2:** Summary of quantitative trait loci (QTL) results from genome-wide association studies (GWAS) of pre-harvest sprouting (PHS) resistance in Chinese wheat landraces.

QTL	Locus	Marker	Mb	Env.	-log10P	R2	Reference
QTL1^#^	3A	AX-111578083	173.81	5	7.23 ∼ 13.95^a^	11.5–25.1%	*Tamyb10-A1*
QTL2^#^	3D	A11134 ∼ A36269	110.99 ∼ 113.89	6	6.58 ∼ 12.74	4.4–19.3%	*Tamyb10-D1*
QTL3^#^	5D	AX-109028892	39.36	3	6.79 ∼ 7.00	11.5–12%	
GCR-QTL1^$^	1A	AX-110008090	0.35	2	9.14 ∼ 10.86	21.8–33.8%	Mohan et al., 2009
GCR-QTL2^$^	1A	AX-94464571	233.53	2	8.75 ∼ 9.68	23.5–30.6%	Singh et al., 2010
GCR-QTL3^$^	1B	AX-109866527	221.04	3	8.62 ∼ 10.1	23–23.6%	Jaiswal et al., 2012
GCR-QTL4^$^	1B	AX-109555236	248.86	2	8.58 ∼ 12.96	19.6–25.7%	
GCR-QTL5^$^	1D	AX-111775865	20.07	2	8.53 ∼ 11.11	19.6–34.3%	
GCR-QTL6^$^	2A	AX-109973468	104.07	3	9.36 ∼ 13.08^a^	21.2–31.2%	Mohan et al., 2009; Jaiswal et al., 2012
GCR-QTL7^$^	2B	AX-94693825 ∼ AX-110478651	102.36 ∼ 118.23	2	8.72 ∼ 11.40	21.4–34.6%	Kumar et al., 2009; Zhang et al., 2014
GCR-QTL8^$^	2B	AX-110645544	160.78	2	10.62 ∼ 12.48	21.5–29.5%	Kumar et al., 2009; Zhang et al., 2014
GCR-QTL9^$^	2B	AX-109008046	209.62	2	9.27 ∼ 11.48	25.6–32.9%	Kumar et al., 2009; Zhang et al., 2014
GCR-QTL10^$^	2B	AX-111478580	296.90	3	8.82 ∼ 10.93	21.9–29.7%	
GCR-QTL11^$^	2B	AX-110610210 ∼ AX-111741521	338.83 ∼ 340.34	3	8.42 ∼ 12.95	22.1–30.1%	
GCR-QTL12^$^	3A	AX-109376167 ∼ AX-111037462	151.44 ∼ 152.19	2	8.81 ∼ 11.67	19.3–35.7%	Rasul et al., 2009
GCR-QTL13^$^	3A	AX-111578083	173.81	2	9.51 ∼ 12.81	22.4–37.1%^b^	*Tamyb10-A1*
GCR-QTL14^$^	3B	AX-111495497	56.99	3	9.35 ∼ 11.6	23.1–31.3%	Jaiswal et al., 2012
GCR-QTL15^$^	3B	AX-110619077 ∼ AX-111560777	121.63 ∼ 126.95	3	8.59 ∼ 13.27^a^	20.7–35.2%	Mares et al., 2009; Chang et al., 2010; Zhang H.P. et al., 2010
GCR-QTL16^$^	3B	AX-108930833	197.07	2	8.51 ∼ 10.11	28.1–29.1%	Mares et al., 2009; Chang et al., 2010; Zhang H.P. et al., 2010
GCR-QTL17^$^	3B	AX-109353822	249.91	2	8.19 ∼ 13.42^a^	19.3–37.3%^b^	Mares et al., 2009; Chang et al., 2010; Zhang H.P. et al., 2010
GCR-QTL18^$^	3B	AX-111819945	286.02	2	8.46 ∼ 8.63	19.3–20.2%	Mares et al., 2009; Chang et al., 2010; Zhang H.P. et al., 2010
GCR-QTL19^$^	3B	AX-110978491	299.58	3	8.59 ∼ 10.86	19.6–27.9%	Mares et al., 2009; Chang et al., 2010; Zhang H.P. et al., 2010
GCR-QTL20^$^	3B	AX-111529538	421.31	2	6.58 ∼ 11.29	15–26.6%	Mares et al., 2009; Chang et al., 2010; Zhang H.P. et al., 2010
GCR-QTL21^$^	3B	AX-109861314 ∼ AX-111106200	494.35 ∼ 505.33	3	8.37 ∼ 13.36^a^	19–36.5%^b^	Jaiswal et al., 2012; Cabral et al., 2014
GCR-QTL22^$^	3B	AX-111194600	767.96	2	8.15 ∼ 8.52	19.4–21.1%	Rehman Arif et al., 2012; Jaiswal et al., 2012
GCR-QTL23^$^	4A	AX-109919526	0.58	2	8.3 ∼ 8.76	21.6–25.1%	
GCR-QTL24^$^	4A	AX-111634210	208.43	3	8.53 ∼ 12.86	19.5–36.4%^b^	Albrecht et al., 2015; Lin et al., 2015; Torada et al., 2016
GCR-QTL25^$^	5A	AX-109844264 ∼ AX-111698406	100.05 ∼ 103.36	2	8.46 ∼ 10.60	21–28.5%	
GCR-QTL26^$^	5B	AX-89623229	19.77	3	8.43 ∼ 9.66	19.1–23.1%	
GCR-QTL27^$^	6A	AX-111007766 ∼ AX-111732156	23.31 ∼ 31.33	2	8.52 ∼ 13.06^a^	19.4–34.9%	
GCR-QTL28^$^	6B	AX-109834362	79.00	3	8.32 ∼ 13.05^a^	20.4–37.4%^b^	
GCR-QTL29^$^	6B	AX-108844376	92.71	2	9.3 ∼ 11.53	22.1–31.6%	
GCR-QTL30^$^	7A	AX-110909277 ∼ AX-111486355	136.77 ∼ 141.52	3	8.27 ∼ 12.8	18.7–36%	
GCR-QTL31^$^	7A	AX-110478067	170.37	3	10.15 ∼ 12.65	24.4–34%	
GCR-QTL32^$^	7B	AX-110932737	102.42	2	9.32 ∼ 12.66	22.3–35.3%	Cabral et al., 2014

*^#^indicates a main QTL identified using all accessions; ^$^indicates a grain color related QTL (GCR-QTL) identified in the red-grained group; ^a^indicates the -log_*10*_P value is higher than 13; ^b^indicates the QTL explained more than 36% of the phenotypic variance*.

Among the three main QTL identified, QTL3 on Chr 5D appears to be novel (**Figure 3D**, **Tables 2**, and Supplementary Table S7) while QTL1 and QTL2 were located in close proximity to known genes or previously reported QTL for grain dormancy or PHS resistance in wheat (**Figures 3B,C** and Supplementary Table S7). QTL1 was positioned close to the *Tamyb10* gene located at 174.1 Mb on Chr 3A which is significantly associated with grain color and germination (Himi et al., 2011; Dong et al., 2015; Lin et al., 2015). SNP maker AX-111578083 linked to QTL1 was positioned only 0.3 Mb away from *Tamyb10* (**Figure 3B**). At least three QTL have been previously mapped within the region spanning 102.3–119.3 Mb on Chr 3D and includes the *R-loci* (Groos et al., 2002; Fofana et al., 2009; Rasul et al., 2009). QTL2 identified in this study (110.99–113.89 Mb, located near the *R-loci* region) also overlaps with major QTL previously reported on Chr 3D (**Figure 3C**). Overall, three main QTL for PHS resistance detected in Chinese wheat landraces were located on Chromosomes 3A, 3D, and 5D.

### Haplotype Analyses for Main QTL Confering PHS Resistance

**FIGURE 4:**
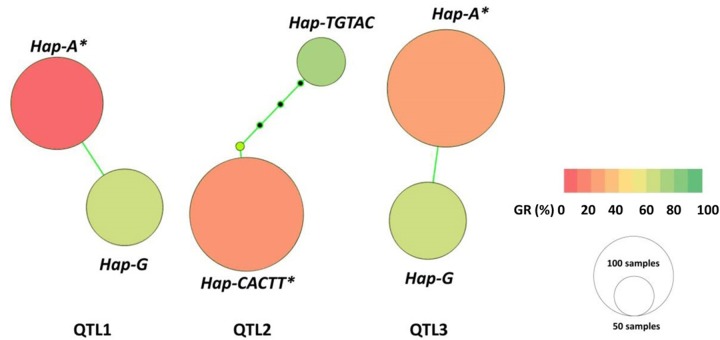
**Haplotype networks for the three main QTL**. The nodes represent variants for the haplotype blocks, with sizes proportional to the number of genotypes carrying the respective variant and colors indicating phenotypic means among genotypes carrying the specific haplotype variant. ^∗^The germination rate for accessions carrying the favorable haplotype is significantly lower than accessions carrying the unfavorable haplotype (*p* < 0.01).

Each main QTL detcted in the panel of 717 landraces (i.e., QTL1, QTL2, and QTL3) had two haplotypes; one favorable and another unfavorable for PHS reisitance. Haplotype A for QTL1 (QTL1-HAP-A) was observed in 71 accessions, where the mean GR ranged from 60.9 to 89.5% across all evironments (**Table 3** and **Figure 4**). Whereas, haplotype G for QTL1 (QTL1-HAP-G) was observed in 109 accessions that showed an average GR of 9.0–38.8% (**Table 3** and **Figure 4**). From the ANOVA results, the GR of accessions from haplotpye G was significantly lower than that of haplotpye A. Therefore, QTL1-HAP-G was defined as the favorable haplotype and QTL1-HAP-A as the unfavorable haplotype for PHS resistance. Haplotype TGTAC in QTL2 (QTL2-HAP-TGTAC) was present in 30 accessions showing an avgerage GR of 67.7–94.2% (**Table 3** and **Figure 4**). On the other hand, haplotype CACTT for QTL2 (QTL2-HAP-CACTT) was observed in 176 accessions showing a significantly lower average GR of 20.1–42.4% (**Table 3** and **Figure 4**). Hence the QTL2-HAP-CACTT was defined as the favorable haplotype and QTL2-Hap-TGTAC as the unfavorable haplotpye for PHS resistance. Haplotpye G for QTL3 (QTL3-HAP-G) was observed in 74 accessions showing an average GR of 59.2–83.2% across the enviornments (**Table 3** and **Figure 4**). In contrast, haplotype A for QTL3 (QTL3-HAP-A) was present in 179 accessions which showed a significantly lower average GR of 21.7–35.5% (**Table 3** and **Figure 4**), hence QTL3-HAP-A was defined as the favorable haplotype and QTL3-HAP-G as the unfavorable haplotpye for PHS resistance.

**Table 3:** Phenotypes (GR) and frequencies of haplotypes (or alleles) for the three main QTL identified via GWAS using all accessions.

Main QTL	Haplotpye	Frequency of Hap	GR 2012 Ya’an	GR 2013 Wenjiang	GR 2014 Wenjiang	GR 2014 Chongzhou	GR 2015 Wenjiang	GR 2015 Chongzhou
QTL1	Hap-A^a^	60.6%	38.8%	15.8%	9.0%	12.6%	10.7%	17.7%
	Hap-G^b^	39.4%	89.5%	85.2%	63.9%	63.8%	60.9%	73.4%
QTL2	Hap-CACTT^a^	85.4%	42.4%	31.3%	20.1%	25.4%	23.5%	29.0%
	Hap-TGTAC^b^	14.6%	94.2%	91.4%	71.6%	67.7%	68.8%	77.1%
QTL3	Hap-A^a^	70.8%	30.1%	32.5%	21.7%	26.2%	24.0%	30.1%
	Hap-G^b^	29.2%	71.8%	83.2%	59.7%	60.7%	59.1%	71.8%

^a^*Favorable haplotpye, ^b^Unfavorable haplotpye determined by ANOVA test*.

### Geographic Distribution of Main QTL for PHS Reisitance

**FIGURE 5:**
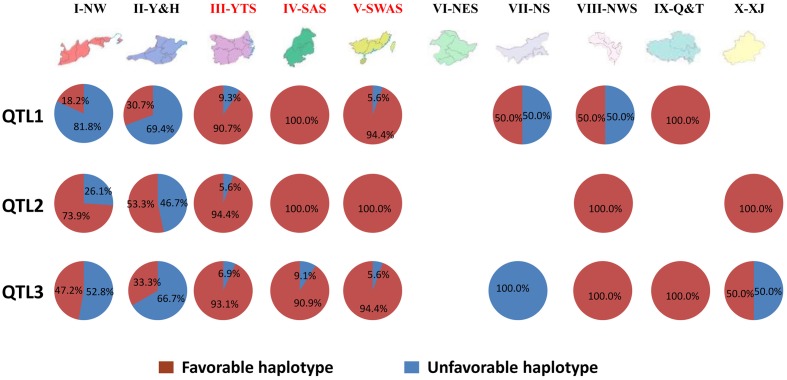
**Geographic distribution of haplotypes for the three main QTL in 10 Chinese Wheat-growing Zones**.

To investigate the geographic distribution of the three main QTL, the frequency of the six haplotypes was determined for landraces sourced from 9 of the 10 Chinese wheat-growing zones (**Figure 5**).

High frequencies (94.4–100%) of the favorable haplotype for QTL1 (QTL1-HAP-G) were observed in landraces from zones III-YTS, IV-SAS, V-SWAS, and IX-Q&T. Half of the accessions originating from zones VII-NS and VIII-NWS carried the QTL1-HAP-G favorable haplotype, while only 18.2 and 30.7% of accessions from zones I-NW and II-Y&H carried it. More than 50% of all accessions carried the favorable haplotype for QTL2 (QTL2-HAP-CACTT) in all test zones. The frequencies of QTL2-HAP-CACTT were 53.3, 73.9, and 94.4% in landraces from zones II-Y&H, I-NW, and III-YTS, respectively. All accessions from zones IV-SAS, V-SWAS, VIII-NWS, and IX-Q&T had the favorable haplotype QTL2-HAP-CACTT. None of the accessions in zone VII-NS carried the favorable haplotype for QTL3 (QTL3-HAP-A). The QTL3-HAP-A favorable haplotype occurred in 33.3, 47.2, and 50.0% of accessions from zones I-NW, II-Y&H, and X-XJ. High frequencies of QTL3-HAP-A were observed in zones III-YTS, IV-SAS, V-SWAS, VIII-NWS, and IX-Q&T. More than 80% of all accessions from zones III-YTS, IV-SAS, V-SWAS, VIII-NWS, and IX-Q&T had all three favorable haplotypes. Overall, the frequency of favorable haplotypes for main QTL contributing PHS resistance in Chinese wheat landraces varied depending on their geographical origin (**Figure 5**).

### Additional Grain Colour Related QTL (GCR-QTL)

To investigate QTL that may have been masked by the main QTL associated with grain color (e.g., QTL1 on 3A and QTL2 on 3D) GWAS was repeated for red- and white-grained accessions separately. Among the 272 accessions that were genotyped using Wheat660 SNP array (Supplementary Table S2), 186 accessions were red-grained, and 77 accessions were white-grained, while 9 accessions didn’t have any color information in Chinese Crop Germplasm Resources Information System.

Firstly, 178,803 polymorphic SNP markers were used for Bayes structure analysis of the two germplasm sets. *K* = 4 and *K* = 2 showed the highest peak in both white- and red-grained accessions, respectively. Next, compressed mixed linear model accounting for the population structure and familial relationship was then used to examine marker-trait assoications within both groups. In the white-grained accessions, no marker was found to be significantly associated with GR (**Figure 3E**). However, in the red-grained group, a total of 46 significant markers obtained –log_10_P values > 8.25 [-log10(0.001/178803)] in at least two environments (**Figure 3F** and Supplementary Table S6). This resulted in the detection of 32 GCR-QTL for PHS resistance within the red-grained sub-group, of which 20 QTL were previously reported, as indicated in **Table 2**. Eleven GCR-QTL were detected in the A genome, 20 GCR-QTL in the B genome, but only 1 GCR-QTL was detected in the D genome. In addition, some markers displayed high –log_10_P values (> 8.25) in only one environment (e.g., AX-110367306, AX-108874755, and AX-109979143), therefore were not selected for further analysis (**Figure 3F**).

**FIGURE 6:**
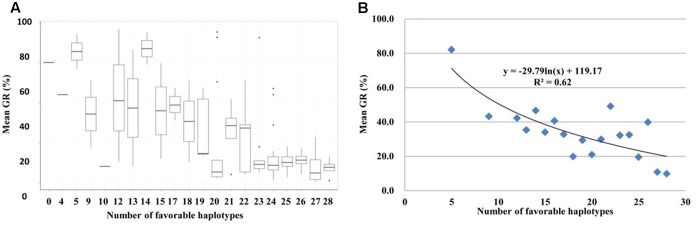
**Pyramiding effect of favorable haplotypes for 32 grain color related QTL (GCR-QTL) in red-grained landrace accessions. (A)** Box plot for GR obtained by accessions grouped according to favorable haplotype numbers. The middle horizontal line within each box represents the mean GR and vertical lines mark the range from 5 to 95% of the total GR within each group. **(B)** Regression analysis with GR and the number of favorable haplotypes.

By determining the presence/absence of the favorable haplotype for each of the 32 GCR-QTL in the 186 red-grained wheat accessions, the total number of favorable haplotypes in each landrace was calculated. Interestingly, a highly significant correlation was observed between the number of favorable haplotypes occurring in landrace accessions and the mean GR obtained across all six environments (*R*^2^ = 0.62; **Figure 6**). Red-grained landraces were highly enriched with favorable haplotypes for GCR-QTL. For instance, 135 of the 186 red-grained landraces carried more than 20 favorable haplotypes.

## Discussion

We have characterized the largest number of wheat landraces for PHS resistance to date – providing new insight into the genetic architecture of this important trait and the geographical distribution of favorable haplotypes across the wheat-growing zones in China.

Genome-wide association studies using germination data collected across six environments identified three main QTL in the collection of 717 landraces, plus 32 GCR-QTL when the red-grained accessions were analyzed separately. However, this is not the first study to report genes/QTL for PHS resistance in Chinese wheat landraces (Zhang H.P. et al., 2010; Zhang et al., 2014, 2017; Wang et al., 2011). For example, QTL located on Chr 4A in the landrace Tuotuomai and QTL on Chr 3A and 3B in the landrace Wanxianbaimaizi are reported to be associated with PHS resistance (Chen et al., 2008; Zhang H.P. et al., 2010). It is clear that Chinese wheat landraces provide a useful source of PHS resistance to develop modern cultivars incorporating PHS resistance.

So far, seven genes associated with PHS have also been cloned in wheat, including: *TaVp1* (Nakamura and Toyama, 2001; Chang et al., 2010, 2011; Wang et al., 2011), *TaMFT* (Nakamura et al., 2011), *TaPHS1* (Liu S. et al., 2013; Liu et al., 2015), *TaSdr* (Zhang et al., 2014, 2017), *TaPm19* (Barrero et al., 2015), *Tamyb10* (Dong et al., 2015; Wang et al., 2016), and *TaMKK3* (Torada et al., 2016). Some of them (i.e., *TaVp1*, *TaMFT*, *TaPHS1*, and *TaSdr*) have been used to test PHS in Chinese cultivars by developing KASP markers (Rasheed et al., 2016). In this study, highly significant main QTL were positioned on the group 3 chromosomes. Positioned in close proximity to the strong signals detected on Chr 3A (QTL1) and Chr 3D (QTL2), is the grain color transcription factor *Tamyb10*, known to be associated with PHS resistance (Groos et al., 2002; Dong et al., 2015). *Tamyb10* is located at the distal region of the long arm of Chr 3A, 3B, and 3D, as reported by Himi and Noda (2005) and Himi et al. (2011). *Tamyb10* is considered a strong candidate for the *R-1* gene, which regulates grain color and SD in wheat (Groos et al., 2002) by regulating both ABA and anthocyanin accumulation (Medina-Puche et al., 2014). Although *Tamyb10-D1* has not been mapped on the ‘Chinese Spring’ survey sequence, it is likely that the strong signal of *R-loci* on Chr 3D in the current study is *Tamyb10-D1*. Recently, a molecular investigation of allelic variation in *Tamyb10* provided information on grain color and GR in Chinese wheat (Wang et al., 2016) and *Aegilops tauschii* (Dong et al., 2015). In this study, two genes associated with grain color (*Tamyb10-A1* and *Tamyb10-D1*) were positioned in close proximity to PHS-resistant QTL (**Figure 3A**). However, a QTL was not detected in the region harboring the B genome ortholog *Tamyb10-B1*, which was detected in U.S. winter wheat (Lin et al., 2016). Two color-related genes showed the strongest signals, thus grain color appears to play an important role in PHS resistance in Chinese landraces (Flintham, 2000; Warner et al., 2000; Himi et al., 2002). In all test environments conducted in this study, the germination level of white-grained accessions was significantly higher than red-grained accessions. While red-grained wheat is generally more resistant to PHS (Probert, 2000; Warner et al., 2000), some white-grained accessions have been reported to display high levels of resistance (Torada and Amano, 2002; Bi et al., 2014). In this study, nine white-grained accessions displayed high levels of PHS resistance and were selected for breeding and further genetic studies.

The main QTL positioned on Chr 5D (QTL3) in this study was considered a novel genomic region potentially harboring loci for PHS resistance. This region seems promising for introgression into white-grained wheat cultivars because it does not co-locate with known genes influencing grain color.

Separate GWAS analyses for white- and red-grained accessions were performed in search for GCR-QTL that may have been masked by main QTL. Although a small number of white-grained accessions displayed PHS resistance, no GCR-QTL was detected within this set. This could be due to population size, as this set only contained 77 accessions. Regardless, the genetic architecture of PHS resistance in the identified white-grained accessions should be subjected to further investigation. When GWAS was carried out for the red-grained accessions, a total of 32 GCR-QTL were detected. Of these regions, almost two-thirds (20 GCR-QTL) have been reported in previous mapping studies. Interestingly, *Tamyb10* in the B and D genomes were not identified, only *Tamyb10* (GCR-QTL13). Although grain color genes contribute to PHS resistance in wheat, there is evidence for genetic factors that are not affected by grain color (Lin et al., 2016). Apart from GCR-QTL13, the remaining 31 GCR-QTL did not co-locate with known genes influencing grain color, thus present good candidates to improve PHS resistance in white-grained wheat. Surprisingly, red-grained landraces were enriched with favorable haplotypes for the GCR-QTL and the number of favorable haplotypes was highly correlated with GR (**Figure 6**). This provided further evidence that GCR-QTL significantly contribute to levels of PHS resistance in red-grained wheat accessions. Further, this highlights the genetic complexity of PHS resistance and the challenge plant breeders face to assemble genotypes incorporating adequate levels of resistance.

Certainly, the process of wheat domestication affected many traits, including SD (Huang et al., 2010a). But following domestication, wheat landraces were cultivated for 1000s of years under diverse eco-geographical conditions prior to modern breeding (Dwivedi et al., 2016; Riaz et al., 2016). Interesting links between the origin and spread of haplotypes associated with agro-climatic traits have been found in sorghum (Morris et al., 2013), rice (Weng et al., 2008; Huang et al., 2010b), and soybean (Zhou et al., 2015). PHS resistance traits and their underlying genes may have been subject to natural and artificial selection performed by farmers in specific environments. In this study, the frequency of favorable haplotypes for PHS resistance QTL varied among landraces originating from the 10 wheat grown zones of China. Favorable haplotypes occurred at high frequencies (92.8–97.0%) in landrace accessions sourced from mesic zones III-YTS, IV-SAS, and V-SWAS (**Figure 4**). Notably, PHS occurs more frequently in these zones compared to zones in northern and north-western China (Jin, 1996; He et al., 2000; Xiao et al., 2002; Yuan et al., 2003; Liu L. et al., 2013). Therefore, it seems PHS resistance was an important trait for crop improvement in southern and eastern China where selective pressure for genes/loci controlling PHS resistance is apparent. The high frequency of favorable haplotypes in landraces originating from high rainfall environments highlights the importance of these haplotypes for future breeding efforts to develop cultivars incorporating PHS resistance.

## Author Contributions

YZ, HT, M-PC, and KD carried out experiments, analyzed the data, and contributed to writing; Z-XC, Z-YL, SG, Y-XL, Q-TJ, X-JL, Z-EP, Y-MW, and Y-LZ carried out experiments and analyzed the data. LH contributed to the analysis and writing for the association mapping; J-RW formulated the questions, designed and carried out experiments, analyzed the data and wrote the manuscript.

## Conflict of Interest Statement

The authors declare that the research was conducted in the absence of any commercial or financial relationships that could be construed as a potential conflict of interest.

## Footnotes

1 http://www.affymetrix.com/catalog/prod850001/AFFY/Axiom%26%23174%3B-Wheat-Genotyping-Arrays#1_3

2 ftp://ftp.ensemblgenomes.org/pub/release-28/plants/fasta/triticum_aestivum/dna/

3 http://popart.otago.ac.nz
